# PReS-FINAL-2289: Ovarian dysfunction in adult childhood-onset lupus patients: a possible role of methotrexate?

**DOI:** 10.1186/1546-0096-11-S2-P279

**Published:** 2013-12-05

**Authors:** DB Araujo, LY Yamakami, E Bonfá, VS Viana, SG Pasoto, RM Pereira, PC Serafin, EF Borba, CA Silva

**Affiliations:** 1 Rheumatology Division, University of São Paulo, São Paulo, Brazil; 2 Gynecology Division, University of São Paulo, São Paulo, Brazil; 3 Pediatric Rheumatology Unit, University of São Paulo, São Paulo, Brazil

## Introduction

Reduction of ovarian reserve has been observed in childhood-onset SLE (c-SLE) and adult SLE populations, and most of them were limited to follicle stimulating hormone (FSH) levels and few recent reports included antral follicle count (AFC) and/or anti-Müllerian hormone (AMH) levels. In addition, the contribution of diminished follicle ovarian pool using anti-corpus luteum antibodies (anti-CoL) was not available in pediatric lupus population.

## Objectives

There are, however, no data regarding the impact of isolated methotrexate exposure and anti-CoL in ovarian reserve of adult c-SLE patients.

## Methods

Fifty-seven adult c-SLE female patients and 21 healthy controls were evaluated for anti-CoL by immunoblot. Complete ovarian function was assessed on the early follicular phase of the menstrual cycle or randomly for those with sustained amenorrhea, blinded to the other parameters of ovarian function. Ovarian reserve was assessed by: FSH, luteinizing hormone (LH), estradiol, AMH and AFC in patients without hormonal contraception for at least 12 consecutive months. Demographic data, menstrual abnormalities, disease activity, damage and treatment were also studied.

## Results

The median of current age was similar in adult c-SLE patients and controls (27.7. vs. 27.7 years, p = 0.414). The median of AMH levels (1.1 vs. 1.5 ng/mL, p = 0.037) and AFC (6 vs. 16, p < 0.001) were significantly reduced in SLE patients *versus *controls without any significant menstrual abnormalities. Anti-CoL was solely observed in SLE patients (16% vs. 0%, p = 0.103) and not associated with demographic data, ovarian reserve parameters, disease activity/damage and treatment. Further evaluation of patients treated with cyclophosphamide revealed a higher median of FSH levels compared to SLE patients not treated with cyclophosphamide and with controls (8.8 vs. 5.7 vs. 5.6 IU/L, p = 0.032) and a lower median AMH levels (0.4 vs. 1.5 vs. 1.5 ng/mL, p = 0.004) and AFC (4.0 vs. 6.5 vs. 16 IU/L, p = 0.001). Nineteen patients were treated with methotrexate without cyclophosphamide use, and a negative correlation was observed between cumulative methotrexate dose and AMH levels (r = -0.507, p = 0.027).

## Conclusion

The present study demonstrated for the first time that high cumulative methotrexate dose is a possible relevant cause of subclinical ovarian dysfunction in adult c-SLE patients and confirms the deleterious effect of cyclophosphamide. These data reinforce the need of gonadal protection during immunosuppressive treatment and fertility counseling.

## Disclosure of interest

D. Araujo: None declared, L. Yamakami: None declared, E. Bonfá: None declared, V. Viana: None declared, S. Pasoto: None declared, R. Pereira: None declared, P. Serafin: None declared, E. Borba: None declared, C. Silva Grant/Research Support from: FAPESP 11/12471-2 to CAS, CNPq 302724/2011-7 to CAS, Federico Foundation to CAS and Núcleo de Apoio à Pesquisa "Saúde da Criança e do Adolescente" da USP (NAP-CriAd) to CAS.

